# OTO-Net: An Automated MRA Image Segmentation Network for Intracranial Aneurysms

**DOI:** 10.1155/2022/5333589

**Published:** 2022-04-14

**Authors:** Jianming Ye, Xiaomei Xu, Liuyi Li, Jialu Zhao, Weiling Lai, Wenting Zhou, Chong Zheng, Xiangcai Wang, Xiaobo Lai

**Affiliations:** ^1^First Affiliated Hospital, Gannan Medical University, Ganzhou, China; ^2^School of Medical Technology and Information Engineering, Zhejiang Chinese Medical University, Hangzhou, China; ^3^Longyan First Affiliated Hospital, Fujian Medical University, Fuzhou, China

## Abstract

Intracranial aneurysms are local dilations of the cerebral blood vessels; people with intracranial aneurysms have a high risk to cause bleeding in the brain, which is related to high mortality and morbidity rates. Accurate detection and segmentation of intracranial aneurysms from Magnetic Resonance Angiography (MRA) images are essential in the clinical routine. Manual annotations used to assess the intracranial aneurysms on MRA images are substantial interobserver variability for both aneurysm detection and assessment of aneurysm size and growth. Many prior automated segmentation works have focused their efforts on tackling the problem, but there is still room for performance improvement due to the significant variability of lesions in the location, size, structure, and morphological appearance. To address these challenges, we propose a novel One-Two-One Fully Convolutional Networks (OTO-Net) for intracranial aneurysms automated segmentation in MRA images. The OTO-Net uses full convolution to achieve intracranial aneurysms automated segmentation through the combination of downsampling, upsampling, and skip connection. In addition, loss ensemble is used as the objective function to steadily improve the backpropagation efficiency of the network structure during the training process. We evaluated the proposed OTO-Net on one public benchmark dataset and one private dataset. Our proposed model can achieve the automated segmentation accuracy with 98.37% and 97.86%, average surface distances with 1.081 and 0.753, dice similarity coefficients with 0.9721 and 0.9813, and Hausdorff distance with 0.578 and 0.642 on these two datasets, respectively.

## 1. Introduction

Intracranial aneurysms are abnormal projections that occur on the walls of cerebral arteries. Such hemorrhage is common among relatively young people, with a higher mortality and incidence rate and about 3% in healthy adults [1]. The main threat comes from subarachnoid hemorrhage (SAH) caused by the rupture of an intracranial aneurysm [2], which accounts for more than 85% [3, 4]. According to the survey, SAH is a catastrophic event, with a mortality rate as high as 25%–60% after rupture [5]. Therefore, accurate measurement and evaluation of the shape of intracranial aneurysms are crucial in daily clinical work so that we can monitor and analyze the growth and rupture risks of aneurysms, making it easier to intervene or treat early [6, 7].

According to the current form of technological development, combined with the actual clinical situation, we found that the inspection methods for intracranial aneurysms include invasive and noninvasive. Compared with invasive Digital Subtraction Angiography (DSA), Computed Tomography Angiography (CTA), magnetic resonance angiography (MRA), and Transcranial Doppler (TCD) have been advocated as the best detection methods for intracranial aneurysms [8]. MRA is a noninvasive angiographic method that does not require radiation exposure. With the introduction of new technologies such as 3D imaging, contrast enhancement, and 3T magnetic field, its sensitivity can reach the detection of aneurysms less than 3 mm [9]. Therefore, based on the simple operation and accurate imaging characteristics of MRA technology, radiologists usually use it to perform 3D visualization and quantitative analysis of small intracranial aneurysms [10]. However, the MRA image of a particular part usually contains part of normal tissue, and it may have a relatively large proportion. Therefore, currently in clinical practice, experts often use manual segmentation to separate the tumor tissue regions. In this context, the segmentation results are greatly affected by the subjective experience of experts, and the repetition rate is low and time-consuming. With the development of emerging technologies such as deep learning, some automated segmentation algorithms have gradually emerged, which have initially solved the problem of manual segmentation. However, the accuracy of these algorithms is still to be discussed.

Under the background of the current era of big data, driven by artificial intelligence, it automatically recognizes complex pattern features in image data and provides quantitative data evaluation results [11]. In particular, the application of Convolutional Neural Network (CNN) [12] in deep learning has shown superior performance in a series of image recognition tasks [13], including medical image processing [14–17]. At present, the research on intracranial aneurysms is not in-depth, but some researchers have made courageous attempts. Park et al. developed and applied a neural network segmentation model called the HeadXNet model, which can generate accurate voxel prediction of intracranial aneurysms on head CTA images [18]. Wang et al. presented a multilevel segmentation method based on the lattice Boltzmann method (LBM) and level set with ellipse for accurate segmentation of intracranial aneurysms, making it potential for clinical assessment of the volume and aspect ratio of the intracranial aneurysms [19]. However, these studies focused on unruptured intracranial aneurysms (UIAs) and did not include patients with Aneurysmal subarachnoid hemorrhage (aSAH). Hence, it remains unclear how deep learning model (DLM) algorithms perform on patients with acutely ruptured intracranial aneurysms (RIAs) and whether the extent of hemorrhage impedes detection sensitivity. Moreover, there still has room for performance improvement due to the significant variability of lesions in the location, size, structure, and morphological appearance.

Based on this, we propose a novel automated MRA image segmentation network for intracranial aneurysms, which can quickly locate intracranial aneurysms and accurately segment its three 3D shapes to help radiologists quantitatively evaluate MRA examinations. The contributions are as follows:

1. A novel 3D image preprocessing scheme is designed to correlate the structural information between data blocks through overlap and partition operations. If the Graphic Processing Unit (GPU) is large enough, the kind of processing is not necessary;
2. A new One-Two-One Fully Convolutional Network (OTO-Net) for intracranial aneurysms automated segmentation in MRA images is proposed, which is based on the idea of fully convolutional networks with three consecutive encoding and decoding structures to detect and segment intracranial aneurysms more efficiently and accurately;
3. Aiming at the severe imbalance of categories caused by small intracranial aneurysms, a loss ensembles objective function is proposed, which improves the segmentation accuracy and dramatically enhances the stability of the backpropagation of the network structure.

After reviewing the state-of-the-art in the field of traditional machine learning-based segmentation methods, deep learning-based methods, and the current intracranial aneurysms segmentation methods in Section 2, we introduce in detail the structure and method of our proposed model in Section 3. Then, we describe the details and results of the experiment in Section 4. Finally, we present a discussion in Section 5 and draw the conclusions in Section 6.

## 2. Related Work

### 2.1. Traditional Machine Learning-Based Segmentation Methods

For a long time in the past, traditional machine learning algorithms have occupied a significant position in image segmentation. Traditional machine learning algorithms include decision tree, random forest, extra tree, ridge classifier, logistic regression, K-Nearest Neighbor [20], Naive Bayes (Gaussian) [21], and Kernel Support Vector Machine (polynomial, Gaussian) [22], and other algorithms. Yang et al. used the random forest method to build a predictive model of cardiovascular disease and achieved significant results, referencing cardiovascular disease prediction and treatment [23]. In addition, Bender used regression models to analyze epidemiological statistics so that an adjusted effect estimate can be obtained that takes into account the impact of potential confounding factors [24]. On the other hand, as the advancement of high-throughput technology had resulted in the generation of a large amount of genomic and epigenome data, the classification features of support vector machines were expanding their applications in cancer genomics, leading to the development of new biomarkers and new drug targets [25].

### 2.2. Deep Learning-Based Segmentation Methods

With the application of image segmentation in biomedical image processing, relevant characteristics of this field are also exposed, such as small sample dataset, high segmentation accuracy, and fast segmentation speed. In order to solve this series of problems, new algorithms such as deep learning were born. However, because CNN loses image details in convolution and pooling, the size of the feature map gradually decreases. It does not provide a good indication of the precise contour of the target body. To solve this problem, Shelhamer et al. proposed a Fully Convolutional Network (FCN) [26], which became the basic framework for semantic segmentation tasks. Most of the subsequent networks are improved on this basis. In 2015, Ronneberger et al. proposed the U-Net structure [27], which has the advantages of supporting a small number of data samples, classifying each pixel and high image segmentation efficiency. In 2016, the V-Net structure was introduced [28], a volume segmentation algorithm based on FCN. Due to its 3D convolution, the introduction of Dice objective function, novel data expansion method, and residual learning, this model showed superior performance in the prostate MRI segmentation task. Especially in recent years, image segmentation has been widely used in medical image processing [29].

### 2.3. Intracranial Aneurysms Segmentation Methods

With the development of modern medical imaging technology, it is possible to understand further the structure, size, and other characteristics of the diseased tissue in a noninvasive way to diagnose the progress of the disease. In 1895, the German physicist Wilhelm Konrad Rontgen discovered X-rays [28, 30], which had opened the door to medical imaging. Medical imaging methods commonly used in clinical practice include X-ray imaging, X-ray computed tomography (CT), emission computed tomography (ECT), magnetic resonance imaging (MRI), etc., and various medical imaging equipment are widely used in hospitals [31]. Based on literature reading and clinical experience, MRA is a relatively good imaging method for diagnosing intracranial aneurysms. We learned that relevant scholars had explored the intracranial aneurysms' automatic segmentation and analysis. However, the accuracy of its segmentation and other indicators cannot be fully guaranteed. This means that aneurysm detection proves to be challenging and time-consuming.

Shahzad et al. [32] developed and evaluated a Deep Learning Model (DLM) to automatically detect and segment aneurysms in patients with aneurysmal subarachnoid hemorrhage (aSAH) on computed tomography angiography. The results prove that this method is highly sensitive and can potentially assist treating physicians in aSAH by providing automated detection and segmentation of aneurysms. Law et al. [33] proposed a novel intensity-based algorithm to segment intracranial vessels and the attached aneurysms, which can handle the low-contrast aneurysmal regions affected by turbulent flows. It is grounded on multirange filters and local variances to extract intensity-based image features. Prior to this, the team also designed a new method based on multirange filters and local variance to segment blood vessels and intracranial aneurysms on PCMRA images that achieves an excellent segmentation effect [34]. Even though there are related research results in this field, there is still no systematic system to solve this problem. This shows that in order to achieve the effect of accurately and efficiently segmenting the tumor area, new methods are being explored continuously.

### 2.4. Our Work

**Figure 1:**
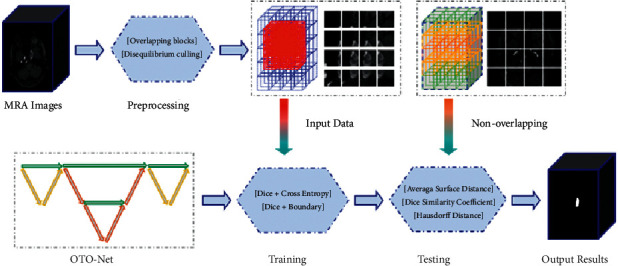
Procedures of our OTO-Net for intracranial aneurysms automated segmentation in MRA images.

Facing the vast challenge of precise segmentation, how to segment the intracranial aneurysm structure from the complex brain tissue structure has become an urgent problem that we need to overcome. Aiming at the characteristics of the existing technology, we proposed a novel model for intracranial aneurysms segmentation in MRA images. We used datasets from the First Affiliated Hospital of Gannan Medical University (GMU) and Aneurysm Detection and segMentation Challenge 2020 (Adam2020, https://adam.isi.uu.nl/). The specific process of our method is as follows: first, the original MRA images of desensitized intracranial aneurysm patients were pretreated. Next, an OTO-Net for intracranial aneurysms automated segmentation in MRA images was proposed. Then, the OTO-Net model was trained on the preprocessed datasets. Finally, the generalization ability of the OTO-Net model is validated by model testing and evaluation indexes. The flow chart of this experimental research is shown in Figure 1.

## 3. Methodology

The purpose of this paper is to realize the accurate and automated segmentation of intracranial aneurysms in MRA images. Therefore, we designed a novel OTO-Net model, which was based on the idea of fully convolutional networks with three consecutive encoding and decoding structures. The process of data preprocessing, OTO-Net network structure, and loss function used were introduced in this section.

### 3.1. Data Preprocessing

MRA image is a 3D data structure, and the 2D network-based segmentation model needs to slice it. The sliced data is 2D data, making the model unable to learn the structural relationship between layers in the data, leading to insufficient network model learning. Therefore, to preserve the spatial expression of intracranial aneurysms, we decided to use 3D data as the model's input. For radiologists' observation and analysis of images, 3D data has significant value in clinical diagnosis and treatment. Below we will elaborate on the 3D image preprocessing method.

#### 3.1.1. Overlapping Blocks

**Figure 2:**
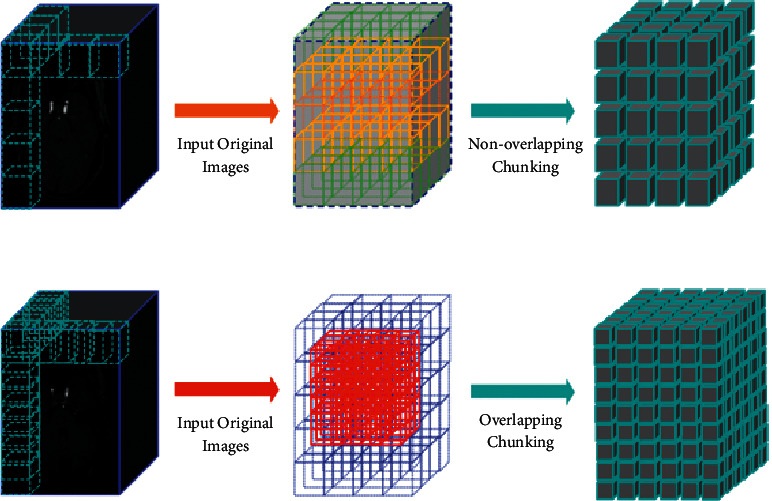
Nonoverlapping chunking and overlapping chunking.

Due to the limitation of GPU memory resources, MRA images cannot be input into the original size network, so the 3D MRA images must be processed in blocks. The paper introduces two chunking methods, namely nonoverlapping block and overlapping block, the structure of which is shown in Figure 2.

The dimension size of the input data of the network structure we designed is 128 × 128 × 64. Here, an MRA image with a size of 512 × 512 × 128 in the dataset is taken as an example. If the same nonoverlapping partitioning processes the original image and the label image, then the step size of each partitioning operation in the *X* and *Y* directions is equal to 128. Furthermore, there is no overlap between the data blocks, so the *X* and *Y* directions are divided into four blocks, respectively. Similarly, if the *Z* step equals 64, it is split into two pieces.

Although 3D data as input solves the structural relationship between the layers of the learning model, nonoverlapping block processing contains up to 64 layers of image information, and the data blocks are still disconnected from each other, making the complete structure unable to be learned. It is similar to the slice image input from the 2D network model. Therefore, we propose an overlapping division method to alleviate this problem. The size of the block remains the same. The step size of the original image and the label image that move simultaneously in the *X*, *Y*, and *Z* directions is smaller than the block size. Although it increases the number of blocks, the advantage is that there is absolute information correlation between blocks.

#### 3.1.2. Disequilibrium Culling

After the block processing, a 3D original image and label image are cut into many 128 × 128 × 64 data blocks. Since the lesion area only occupies a small part of the brain, the block processing partnership will contain the nonlesion blocks. If these data blocks are used together as input data for the model, the class imbalance will be caused, and the model's learning process will be troubled.

Therefore, to mitigate category imbalance in this study, the label image data block is further screened after the chunking process. If there is no data more significant than 0 in the data block, the lesion area is not included, the original image and label image data block would be discarded.

### 3.2. Model Design

**Figure 3:**
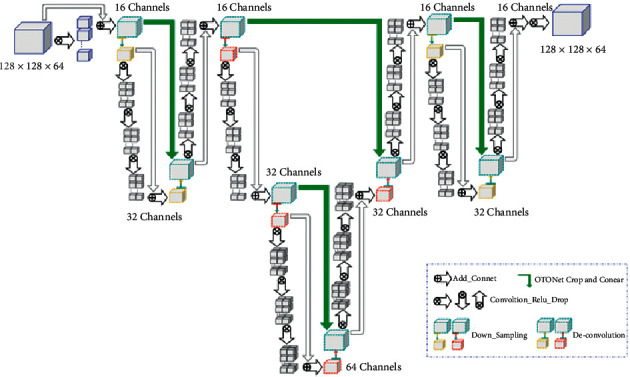
Overall architecture of our proposed OTO-Net.

The OTO-Net is a model based on 3D fully convolutional networks. Its main structure takes advantage of the encoder-decoder proposed by Hinton et al. [35] in 2006. The most significant feature of the OTO-Net structure is the continuous use of three complete encoding and decoding structures to achieve the purpose of accurate detection and segmentation of intracranial aneurysms. The whole network still presents an entirely symmetrical design. The schematic diagram of OTO-Net is shown in Figure 3.

In the OTO-Net structure designed by us, the convolution kernel uses 3 × 3 × 3 voxels, assigns 'SAME′ mode to supplement data, and selects ReLU as the activation function. The three complete encoding and decoding structures in OTO-Net are used in downsampling, upsampling, and skip connection. First, the input data of 64 × 128 × 128 × 1 was received. And then, the convolution check with 3 × 3 × 3 voxels with the convolution step of [1] was performed for two convolution operations. Finally, Layer 1 was obtained by adding the first and second convolution layers.

After being downsampled with a convolution kernel step size of [1, 2], the network structure of Layer1 changes from 16 channels to 32, and then, perform three successive convolution operations and add the downsampled layer and the third convolutional layer to get Layer2. Subsequently, deconvolve Layer2. Moreover, the convolution kernel size was still [1, 2], so the channels changed from 32 to 16. After skip connection between the deconvolution layer and Layer1 stack operation, three consecutive convolution operations and the deconvolution layer are added to obtain Layer3, whose size is the same as Layer1. At this point, the first complete encoding and decoding structure is completed.

Except for the second encoding and decoding structure is different from the first structure, two consecutive downsampling and then two successive upsampling are used, while the third is the same as the first structure. When the OTO-Net structure enters Layer 9, the layer's size is the same as that of Layer1 again. Finally, it passes through the output layer's convolution operation and uses the Sigmoid activation function to map out the prediction result of the size of 64 × 128 × 128 × 1. The theoretical calculation of network parameters of the OTO-Net structure is shown in Table 1.

### 3.3. Loss Ensembles

The loss function, also known as the objective function, solves and evaluates the model by minimizing it, so it is crucial for model construction. Medical images usually have a characteristic that the target area to be segmented is unbalanced with the background area. To solve this problem, Milletari et al. proposed a new objective function in the V-Net structure, which had an immediate effect because the Dice coefficient was cited [36]. Since then, Dice loss has become one of the most critical evaluation indexes of medical image segmentation.

**Figure 4:**
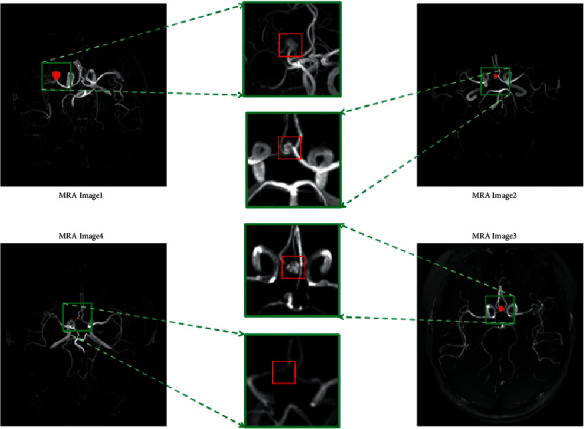
Morphology of intracranial aneurysm at high magnification.

Nevertheless, as Figure 4 shows, intracranial aneurysms account for a tiny proportion of the entire brain, and the foreground and the background are highly imbalanced. Therefore, we propose an objective function optimization for loss ensembles, including the comparative experiment between Dice loss + Cross-Entropy loss and Dice loss + Boundary loss. The principles of each are described in detail in the following sections.

#### 3.3.1. Dice Loss

Dice coefficient is a set similarity measurement function, which is usually used to calculate the similarity between two samples, and its value ranges between [0, 1]. The formula is as follows:

(1) $$\begin{array}{c} s=\frac{2\left|A\cap B\right|}{\left|A\right|+\left|B\right|}, \end{array}$$

where |**A**∩**B**| represents the intersection of set **A** and set **B**, |**A**| and |**B**| represents the number of elements of set **A** and set **B**, respectively. And the coefficient 2 in the numerator is to eliminate the common component of double calculation in the denominator. For semantic segmentation, *p* represents the predicted image and *g* represents the label image. Thus, Dice loss can be expressed as follows:

(2) $$\begin{array}{c} L_{dice}=1-\frac{\left(2\sum_{i=1}^{N}p_{i}g_{i}\right)+w}{\left(\sum_{i=1}^{N}p_{i}^{2}+\sum_{i=1}^{N}g_{i}^{2}\right)+w}, \end{array}$$

where *w* is known as the smoothing coefficient, a minimal number. And it is used to prevent the denominator from being 0. Although Dice loss has a good performance in the scenario where there is a severe imbalance between positive and negative samples and focuses more on the mining of foreground area in the training process, it also has disadvantages. Loss is prone to be unstable, especially in small targets, which will lead to gradient saturation in extreme cases. Therefore, we propose a loss ensembles optimization method to solve these problems.

#### 3.3.2. Cross-Entropy Loss

Suppose two different probability distributions, *P*(*x*) and *Q*(*x*), for the same random variable *X*. In that case, we can use relative entropy (KL divergence) [37] to measure the difference between these two probability distributions. The formula is expressed as follows:

(3) $$\begin{array}{c} D_{KL}\left(P\left\|\right)Q\right)=\sum_{i=1}^{N}P\left(x_{i}\right)\log\left(\frac{P\left(x_{i}\right)}{Q\left(x_{i}\right)}\right), \end{array}$$

(4) $$\begin{array}{c} D_{KL}\left(P\left\|\right)Q\right)=-H\left(P\left(x\right)\right)+\left[-\sum_{i=1}^{N}P\left(x_{i}\right)\log\left(Q\left(x_{i}\right)\right)\right]. \end{array}$$

KL divergence formula (4) can be obtained by further deformation from (3), where the former represents information entropy and the latter represents cross-entropy, that is, KL divergence = cross-entropy − information entropy.

In the model training process, since the input data and labels have been determined, and the true probability distribution *P*(*x*) has also been determined, the information entropy is constant. In addition, since the value of KL divergence represents the difference between the true probability distribution *P*(*x*) and the predicted probability distribution *Q*(*x*), the smaller the value, the better the prediction result. Therefore, it is necessary to minimize the KL divergence to obtain the best results. It is easier to calculate using the cross-entropy loss function as the objective function. Replace *P*(*x*) with *g*(*x*) and *Q*(*x*) with *Q*(*x*). The formula of cross-entropy loss can be expressed as follows:

(5) $$\begin{array}{c} L_{ce}=-\sum_{i=1}^{N}g\left(x_{i}\right)\log\left(p\left(x_{i}\right)\right), \end{array}$$

where the former represents the KL divergence, and the latter is a constant.

#### 3.3.3. Boundary Loss

Boundary loss is an objective function proposed by Kervadec et al. to solve the difficulty of highly unbalanced segmentation [38]. The core of the method is to calculate the distance between the predicted image and the boundary of the marked image through calculus. The calculation formulas are as shown in (6) and (7), respectively:

(6) $$\begin{array}{c} \text{Dist}\left(\partial G\;,\partial S\right)=\int_{\partial G}{\left\|y_{\partial S}\left(p\right)-p\right\|}^{2}dp, \end{array}$$

(7) $$\begin{array}{c} \text{Dist}\left(\partial G\;,\partial S\right)\approx 2\int_{\Delta s}D_{G}\left(q\right)dp. \end{array}$$

Since the distance differential expression (6) cannot be used as a loss function, the integral expression (7) is adopted. The crucial region △*S* is divided into *S* and *G*, and the binary indicators function *s*(*q*) and *g* (*q*) about segmentation and ground truth are introduced so that the integral region extends from *S* and *G* to the entire image domain.

(8) $$\begin{array}{c} \Delta S=\left(\frac{S}{G}\right)\cup \left(\frac{G}{S}\right), \end{array}$$

(9) $$\begin{array}{c} \frac{1}{2}\text{Dist}\left(\partial G\;,\partial S\right)=\int_{S}\phi G\left(q\right)dq-\int_{G}\phi G\left(q\right)dq \end{array}$$

Then we replaced *s*(*q*) in formula (9) with *S*(*q*), which established a connection with the softmax output of the network. At this point, we can obtain the Boundary loss function expression (11):

(10) $$\begin{array}{c} \underset{\theta }{\min}Di\;st\left(\partial G\;,\partial S_{\theta }\right)\approx \int_{\Omega }\phi G\left(q\right)S_{\theta }\left(q\right)dq-\int_{\Omega }\phi G\left(q\right)g\left(q\right)dq, \end{array}$$

(11) $$\begin{array}{c} L_{B}\left(\theta \right)=\int_{\Omega }\phi G\left(q\right)S_{\theta }\left(q\right)dq. \end{array}$$

## 4. Experiments

### 4.1. Datasets

In the study, we evaluated the proposed OTO-Net on one public benchmark dataset and one private dataset used to train, evaluate and objectively compare the performance of standardized intracranial aneurysm MRA data segmentation algorithms. First, we use the Adam2020 dataset. A set of 255 representative intracranial aneurysm MRA data is shared on the challenge website (https://adam.isi.uu.nl/) [2]. Adam2020 dataset includes 113 training data and 142 test data, and about 32 epochs are needed for training the model. In addition, the second in-house private dataset is provided by the First Affiliated Hospital of Gannan Medical University (GMU dataset). In total, 65 clinical intracranial aneurysm MRA data are provided with manual segmentation labels to verify the model, including 30 training data and 35 test data. It takes about 27 epochs to train the proposed OTO-Net with GMU dataset.

### 4.2. Evaluation Metrics

In order to have a more systematic evaluation of the effect of segmentation, we have stipulated the following indicators as the evaluation metric. The Average Surface Distance (ASD) refers to the average surface distance of all points in the 3D data block. It is a commonly used evaluation index in medical image segmentation tasks, and its mathematical definition can be expressed by the following formula:

(12) $$\begin{array}{c} ASD=\frac{\sum_{a\in S\left(A\right)}\underset{b\in S\left(B\right)}{\min}\left\|a-b\right\|+\sum_{b\in S\left(B\right)}\underset{a\in S\left(A\right)}{\min}\left\|b-a\right\|}{\left|S\left(A\right)\right|+\left|S\left(B\right)\right|}. \end{array}$$

Next, since Dice Similarity Coefficient (DSC) is equivalent to equation (1), and the sum of Dice loss and DSC is 1. Based on the above conditions, DSC can be expressed as follows by the following formula:

(13) $$\begin{array}{c} \text{DSC}=\frac{2\sum_{i=1}^{N}p_{i}g_{i}}{\sum_{i=1}^{N}p_{i}^{2}+\sum_{i=1}^{N}g_{i}^{2}}. \end{array}$$

If DSC is sensitive to internal filling, Hausdorff distance (HD) focuses on calculating segmentation boundaries. Hausdorff distance is a maximum function describing set A to set B. And the general definition can be expressed by the following formula:

(14) $$\begin{array}{c} \text{HD}\left(C,D\right)=\max\left\{h\left(C,D\right),h\left(D,C\right)\right\}, \end{array}$$

where *h*(*C*, *D*) is called the directed Hausdorff distance and given by *h*(*C*, *D*)=max_*a*∈*C*_min_*b*∈*D*_‖*a* − *b*‖, where ‖*a* − *b*‖ is some norm. In all HD calculations, the maximum distance quantile is set to 95%. The purpose is to eliminate the little distance caused by some outliers to ensure the overall value's stability.

### 4.3. Experimental Details

We use the V-Net model and the OTO-Net model for comparative experiments. As mentioned in the previous article, we used the comprehensive objective function of Dice loss + Cross-entropy loss and Dice loss + Boundary loss in the training process. The intracranial aneurysm segmentation study provided two datasets, so we designed Table 2 that shows the experimental protocol.

### 4.4. Training Process

As shown in Table 3, to meet the experimental conditions of the control group without limiting the optimal performance of the model, we reasonably set the training parameters of each group of experiments, such as learning rate, dropout, epoch, and batch size.

In this study, we used the python-based PyCharm framework to implement the preprocessing and our proposed OTO-Net model, including the software packages such as 1.19.2 NumPy, 3.1.1 Nibabel, 2.0.0 SimpleITK, 1.1.2 Pandas, 4.4.0 OpenCV, and the GPU version of 1.13.1 TensorFlow. The workstation is installed with the Window10 system, equipped with two Intel(R) Xeon(R) Silver 4210 CPU @2.20 GHz, one NVIDIA TITAN RTX 24 GB GPUs, and 128G running memory. All different modes were tested under the same GPU and environment.

## 5. Results and Discussion

### 5.1. Results

After completing the model's training process, we tested its segmentation detection performance. Evaluation indicators can be considered as the most intuitive manifestation of results. As shown in Table 4, we used the ASD, DSC, and HD three indicators introduced in the previous article to compare and evaluate the effects of all experimental groups. The last column of the table—comprehensive ranking is the cumulative sum of the scales of each group in the three indicators. It can be implied by its definition that the smaller the value, the higher the overall performance of the corresponding model.

By observing and comparing the experimental data, it can be concluded that Ex3, Ex5, and Ex12 rank first in the three indicators of ASD, HD, and DSC, respectively, and Ex12 is the best in the overall ranking. Combined with the experimental model design, they use all OTO-Net. Therefore, we have reason to believe that O's segmentation effect on V has been further improved. In addition, for another experimental variable loss function, we found that the overall rankings of Ex1, Ex4, Ex7, and Ex10 that only use the dice objective function are relatively low, and the loss ensembles play a critical role in improving the segmentation performance.

We comprehensively analyze and calculate the results of 6 sets of experiments and finally conclude that the accuracy of the OTO-Net model on the GMU and Adam2020 datasets is 98.37% and 97.86%, respectively. The accuracy is the ratio of the correct prediction area to the target area. Ex5 ranked first in the GMU dataset, with ASD reaching 1.989, DSC reaching 0.9721, and HD reaching 0.578 mm; Ex12 ranked first on the Adam2020 dataset with ASD of 0.753, DSC of 0.9813, and HD of 0.642 mm. We have noticed that this model performs better on the Adam2020 dataset and believe that the reason is that the data contained in it is more standardized.

For the clinician's diagnosis, the most intuitive and effective way of presentation is the visualization of the segmentation results. Both OTO-Net and V-Net mentioned in this article have end-to-end characteristics, so the output data size is still 128 × 128 × 64. To preserve the predicted results in the model training process, we converted the 128 × 128 × 64 3D data blocks into 64 128 × 128 2D images and then merged them into 8 × 8 images. The smaller the inference cycle of the model is, the faster the inference speed is, and the stronger the model's performance is. When the reasoning cycles are the same, the reasoning cycles' size can be evaluated by comparing the value of dice loss, and then the performance of the model can be judged.

**Figure 5:**
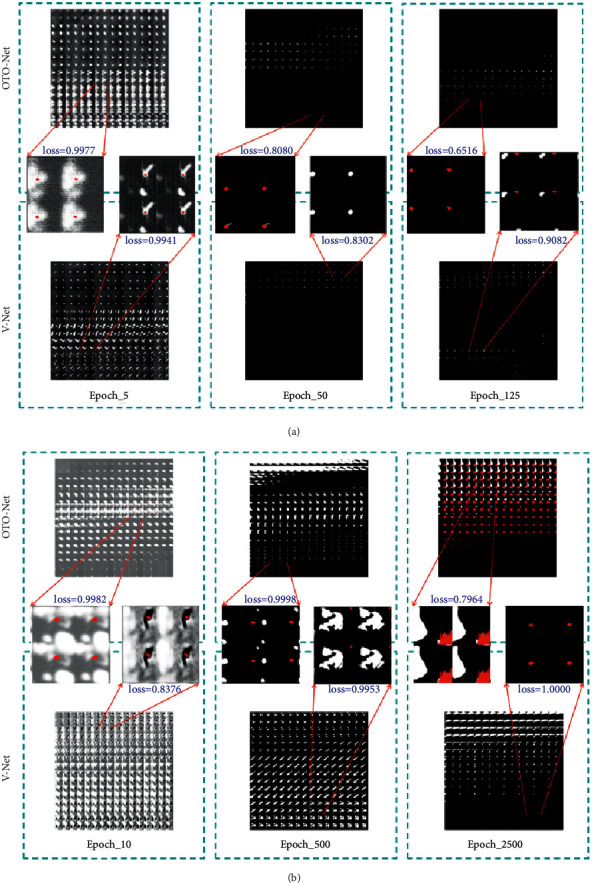
Prediction results comparison between the OTO-Net and V-Net: (a) GMU dataset, (b) Adam2020 dataset.

As shown in Figure 5(a), (a) is the comparison between the prediction results of OTO-Net and V-Net on the GMU dataset in the 5th, 50th, and 125th rounds of the inference period and the standard; (b) is the comparison between the prediction results of OTO-Net and V-Net on the Adam2020 dataset in the 10th, 500^th^, and 2500th rounds of the inference period and the standard. By comparing the four groups of data, it can be found that the dice loss values of Ex5 and Ex12 are significantly smaller, indicating that their predicted results are more similar to the standard. Therefore, the model structure of Ex5 and Ex12 is superior.

**Figure 6:**
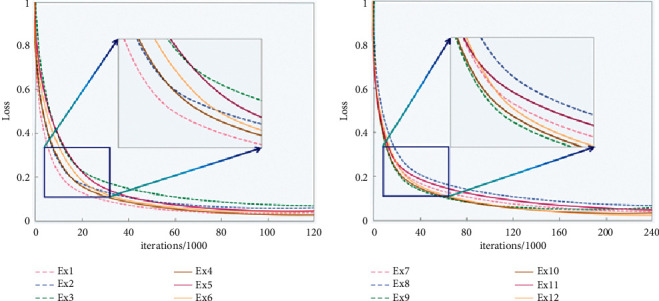
Training loses per group of experiments on GMU dataset and Adam2020 dataset.

On this basis, to observe the loss values of each group of experiments in the training process, Figures 6(a) and 6(b) recorded and described the dynamic change curves of training loss of 12 groups of experiments GMU and Adam2020 datasets, respectively. The volatility of the loss curve is one of the important indexes to measure the model's stability. As shown in Table 5, by calculating the residual sum of squares (RSS) of each loss curve, the volatility of the loss's discrete value was characterized by a quantity that measured the degree of model fit in the linear model. The smaller the matter is, the smaller the fluctuation is, which means that the model tends to be more stable. By comparing the data in the table, it can be seen that the RSS values of Ex4∼6 and Ex10∼12 are small, so the model structure is more stable.

To sum up, all the experimental data, respectively, reflect the excellent effects of training stability, segmentation accuracy, and generalization ability, which effectively verify the advancement of the OTO-Net structure and loss ensembles proposed by us.

### 5.2. Discussion

**Figure 7:**
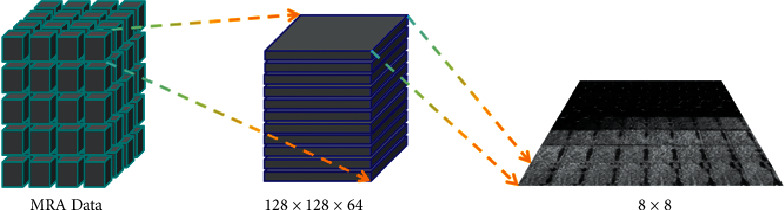
Test set nonoverlapping block processing.

After introducing the process and results of this experiment, it can be seen that OTO-Net with loss ensembles shows high overall accuracy and robustness in the segmentation of highly unbalanced intracranial aneurysms. As shown in Figure 7, the test samples divided in advance by GMU and Adam2020 datasets were first processed by nonoverlapping block processing, respectively, to obtain multiple test datasets with the size of 128 × 128 × 64, and then input them into OTO-Net, respectively, for prediction.

**Figure 8:**
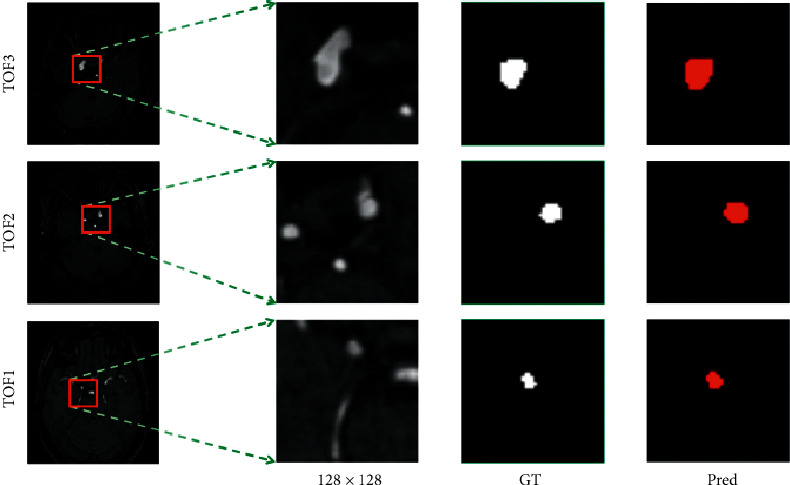
Visualization results of the intracranial aneurysms segmentation using the proposed OTO-Net on GMU dataset.

**Figure 9:**
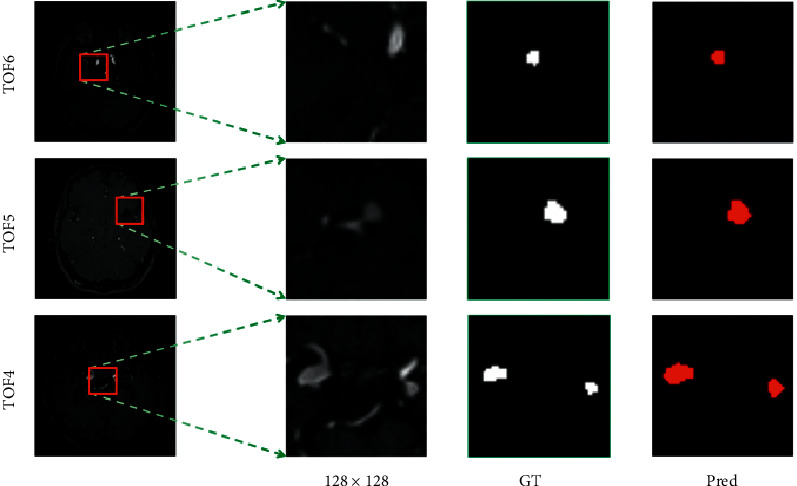
Visualization results of the intracranial aneurysms segmentation using the proposed OTO-Net on Adam2020 dataset.

Figures 8 and 9 respectively, show the three groups of MRA image results predicted by the OTO-Net model on the GMU dataset and the Adam2020 dataset. Because the intracranial aneurysm was too small, we enlarged and clipped the target region to have a precise observation of each result. Observation of the predicted results shows that OTO-Net can still achieve pixel-level segmentation requirements, but for tiny intracranial aneurysms, it is difficult for OTO-Net to achieve absolute precision segmentation in some marginal areas. In order to quantitatively discuss the prediction results, three indicators of ASD, DSC, and HD of the six groups of test data were counted, respectively. As shown in Table 6, ASD of TOF2 reached 0.699 and HD of TOF3 reached 0.506 mm, both of which were higher than the maximum values of the training results. The maximum value of DSC is 0.9803, which is slightly inferior to the maximum value of training.

In order to increase the persuasiveness of the results of the article, we must effectively compare it with the results of previous studies. However, by reading a large number of documents, we can see that few studies overlap entirely with the research field of this article, which fully proves that the idea of the article is quite innovative. The model proposed by Sichtermann et al. has a system with a maximum overall accuracy of 90% for detecting intracranial aneurysms and an accuracy of 96% for aneurysms with a diameter of 3–7 mm, which is lower than the segmentation result obtained by this model [39].

Therefore, it can be seen from the whole that the current research results show that OTO-Net has a high level of segmentation accuracy for large, medium, and small regions and multiregions. OTO-Net is fully competent to assist radiologists in quantitative analysis and evaluation of MRA examination in patients with intracranial aneurysms. Accuracy is the most important index in the field of medical image processing. There is no upper limit to this index, so our current research needs to be more in-depth and detailed.

## 6. Conclusion

This study proposed the OTO-Net model for intracranial aneurysms automated segmentation in MRA images and performed experiments on the Adam2020 dataset and GMU dataset, respectively. We designed a novel 3D image preprocessing scheme to correlate the structural information between data blocks through overlap and partition operations. At the same time, we proposed the OTO-Net for intracranial aneurysms automated segmentation in MRA images. The OTO-Net uses full convolution to achieve intracranial aneurysms automated segmentation through the combination of downsampling, upsampling, and skip connection. In addition, loss ensemble is used as the objective function to steadily improve the backpropagation efficiency of the network structure during the training process. We evaluated the proposed OTO-Net on one public benchmark dataset and one private dataset. Our proposed model can achieve the automated segmentation accuracy with 98.37% and 97.86%, average surface distances with 1.081 and 0.753, dice similarity coefficients with 0.9721 and 0.9813, and Hausdorff distance with 0.578 and 0.642 on these two datasets, respectively. Therefore, our proposed OTO-Net plays an essential role in radiologists' assisted discovery and diagnosis of intracranial aneurysms and brings substantial value to intelligent medicine's advancement and development. The next research direction is to better use the transformer and 3D information to segment intracranial aneurysms in MRA images.

## Figures and Tables

**Table 1:** Parameter details of the proposed OTO-Net.

Layer	Input size	Kernel
O^I^-Stage1	128	3 × 3 × 3 × 16 × 16
O^I^-Stage2	64	3 × 3 × 3 × 32 × 32
T-Stage1	128	3 × 3 × 3 × 16 × 16
T-Stage2	64	3 × 3 × 3 × 32 × 32
T-Stage3	32	3 × 3 × 3 × 64 × 64
T-Stage4	64	3 × 3 × 3 × 32 × 32
T-Stage5	128	3 × 3 × 3 × 16 × 16
O^II^-Stage1	64	3 × 3 × 3 × 32 × 32
O^II^-Stage2	128	3 × 3 × 3 × 16 × 16

**Table 2:** Experimental design of image segmentation for intracranial aneurysms.

Experiment	Dataset	Algorithm	Loss function
Ex1	GMU	V-Net	Dice
Ex2	GMU	V-Net	Dice + cross-entropy
Ex3	GMU	V-Net	Dice + boundary
Ex4	GMU	OTO-Net	Dice
Ex5	GMU	OTO-Net	Dice + cross-entropy
Ex6	GMU	OTO-Net	Dice + boundary
Ex7	ADAM2020	V-Net	Dice
Ex8	ADAM2020	V-Net	Dice + cross-entropy
Ex9	ADAM2020	V-Net	Dice + boundary
Ex10	ADAM2020	OTO-Net	Dice
Ex11	ADAM2020	OTO-Net	Dice + cross-entropy
Ex12	ADAM2020	OTO-Net	Dice + boundary

**Table 3:** The hyperparameters of each experiment.

Experiment	Inputable data	Learning rate	Dropout	Epoch	Batch size
Ex1	597	0.001	0.5	200	4
Ex2	597	0.001	0.5	200	4
Ex3	597	0.001	0.5	200	4
Ex4	597	0.001	0.5	200	2
Ex5	597	0.001	0.5	200	2
Ex6	597	0.001	0.5	200	2
Ex7	1613	0.001	0.5	150	4
Ex8	1613	0.001	0.5	150	4
Ex9	1613	0.001	0.5	150	4
Ex10	1613	0.001	0.5	150	2
Ex11	1613	0.001	0.5	150	2
Ex12	1613	0.001	0.5	150	2

**Table 4:** Evaluation index and a comprehensive score of each experimental model.

Experiment	Time (h)	ASD	DSC	95% HD	Comprehensive ranking
Ex1	11.5	3.562 ± 0.77	0.9391 ± 0.023	7.332 ± 2.31	12/12 + 11/12 + 12/12 = 2.91
Ex2	11.7	2.409 ± 0.26	0.9502 ± 0.032	3.053 ± 1.11	9/12 + 10/12 + 8/12 = 2.25
Ex3	12.1	**0.706** ± **0.05**	0.9672 ± 0.020	2.751 ± 1.29	1/12 + 7/12 + 7/12 = 1.25
Ex4	10.4	2.014 ± 0.20	0.9701 ± 0.017	1.242 ± 2.68	7/12 + 5/12 + 6/12 = 1.50
Ex5	11.0	1.989 ± 0.62	0.9721 ± 0.005	**0.578** ± **0.43**	6/12 + 3/12 + 1/12 = 0.83
Ex6	11.3	1.081 ± 0.27	0.9710 ± 0.013	0.942 ± 0.87	4/12 + 4/12 + 3/12 = 0.91
Ex7	43.6	3.194 ± 0.31	0.9362 ± 0.020	7.116 ± 9.84	11/12 + 12/12 + 11/12 = 2.83
Ex8	43.9	2.113 ± 0.09	0.9586 ± 0.031	5.583 ± 1.92	8/12 + 9/12 + 10/12 = 2.25
Ex9	44.1	2.991 ± 0.31	0.9621 ± 0.017	1.131 ± 0.31	10/12 + 8/12 + 5/12 = 1.91
Ex10	39.8	1.953 ± 0.71	0.9699 ± 0.042	4.112 ± 0.94	5/12 + 6/12 + 9/12 = 1.66
Ex11	40.1	0.905 ± 0.11	0.9761 ± 0.007	1.041 ± 0.31	3/12 + 2/12 + 4/12 = 0.75
Ex12	40.6	0.753 ± 0.03	**0.9813** ± **0.008**	0.642 ± 0.17	**2/12** + **1/12** + **2/12** = **0.33**

**Table 5:** RSS calculation results for each loss curve.

	Ex1/Ex7	Ex2/Ex8	Ex3/Ex9	Ex4/Ex10	Ex5/Ex11	Ex6/Ex12
Residual sum of squares (RSS)	17.7597	17.0351	18.1124	15.9172	**15.1319**	17.0053
	19.8024	19.0271	21.9512	16.3814	18.7011	**16.1217**

**Table 6:** Evaluation indexes of six MRA image test sets.

Test	Dataset	ASD	DSC	95% HD
TOF1	GMU	0.982	0.9604	0.812
TOF2	GMU	**0.699**	0.9711	0.640
TOF3	GMU	0.704	0.9622	**0.506**
TOF4	ADAM2020	1.105	0.9730	0.618
TOF5	ADAM2020	0.786	**0.9803**	0.574
TOF5	ADAM2020	1.032	0.9705	0.713

## Data Availability

The raw/processed data required to reproduce these findings cannot be shared at this time as the data also forms part of an ongoing study.
